# Placebo Responses in Genetically Determined Intellectual Disability: A Meta-Analysis

**DOI:** 10.1371/journal.pone.0133316

**Published:** 2015-07-30

**Authors:** Aurore Curie, Kathy Yang, Irving Kirsch, Randy L. Gollub, Vincent des Portes, Ted J. Kaptchuk, Karin B. Jensen

**Affiliations:** 1 Department of Psychiatry, Massachusetts General Hospital/Harvard Medical School, Martinos Center for Biomedical Imaging, Boston, Massachusetts, United States of America; 2 L2C2, Institut des Sciences Cognitives, CNRS UMR5304, Bron, France; 3 Centre de Référence Déficiences Intellectuelles de Causes Rares, Hôpital Femmes Mères Enfants, Hospices Civils de Lyon, Bron, France; 4 Université Claude Bernard Lyon1, Lyon, France; 5 EPICIME-CIC1407/INSERM, Bron, France; 6 Program in Placebo Studies, Beth Israel Deaconess Medical Center/Harvard Medical School, Boston, Massachusetts, United States of America; 7 School of Psychology, Plymouth University, Plymouth, United Kingdom; 8 Department of Clinical Neuroscience, Karolinska Institute, Stockholm, Sweden; CNRS UMR7275, FRANCE

## Abstract

**Background:**

Genetically determined Intellectual Disability (ID) is an intractable condition that involves severe impairment of mental abilities such as learning, reasoning and predicting the future. As of today, little is known about the placebo response in patients with ID.

**Objective:**

To determine if placebo response exists in patients with genetically determined ID.

**Data sources and Study selection:**

We searched Medline/PubMed, EMBASE, CENTRAL and PsycINFO to find all placebo-controlled double-blind randomized clinical trials (RCTs) in patients with genetically determined ID, published up to April 2013, focusing on core ID symptoms.

**Data extraction and synthesis:**

Two investigators extracted outcome data independently.

**Main outcomes and measures:**

Bias-corrected standardized mean difference (Hedge’s *g*) was computed for each outcome measure, using the Comprehensive Meta-Analysis software. A priori defined patient sub-groups were analyzed using a mixed-effect model. The relationship between pre-defined continuous variable moderators (age, IQ, year of publication and trial duration) and effect size was analyzed using meta-regression

**Results:**

Twenty-two placebo-controlled double-blind RCTs met the inclusion criteria (n = 721, mean age = 17.1 years, 62% men, mean trial duration = 35 weeks). There was a significant overall placebo response from pre- to post-treatment in patients with ID (*g* = 0.468, p = 0.002), both for “subjective outcomes” (a third-person’s evaluation of the patient) (*g* = 0.563, p = 0.022) and “objective outcomes” (direct evaluation of the patient’s abilities) (*g* = 0.434, p = 0.036). Individuals with higher IQ had higher response to placebo (p = 0.02) and no placebo response was observed in ID patients with comorbid dementia. A significant effect of age (p = 0.02) was found, indicating higher placebo responses in treatment of younger patients.

**Conclusions and relevance:**

Results suggest that patients with genetically determined ID improve in the placebo arm of RCTs. Several mechanisms may contribute to placebo effects in ID, including expectancy, implicit learning and “placebo-by-proxy” induced by clinicians/family members. As the condition is refractory, there is little risk that improvements are explained by spontaneous remission. While new avenues for treatment of genetically determined ID are emerging, our results demonstrate how contextual factors can affect clinical outcomes and emphasize the importance of being vigilant on the role of placebos when testing novel treatments in ID.

## Introduction

Intellectual Disability (ID) is characterized by deficits in intellectual functions, such as reasoning, abstract thinking, judgment and learning from experience. The defining features of ID are confirmed by clinical assessment and standardized intelligence testing (overall Intelligence Quotient (IQ)<70), combined with deficits in adaptive functioning that result in failure to meet developmental and sociocultural standards, manifested during the developmental period (DSM-V) [1]. Genetic causes of ID include visible chromosomal anomalies (such as Down’s syndrome, which is the most frequent aneuploidy), chromosomal microdeletion (including Prader-Willi and Williams’ syndrome) and monogenic diseases (such as mutation of the gene *FMR1* leading to Fragile X syndrome). None of these disorders improve spontaneously over time. If anything, patients with Down’s syndrome risk an age-related cognitive decline after 40, which progresses to Alzheimer’s-like dementia [2]. Until recently, treatment for ID focused mainly on symptom management (including attention deficits and anxiety), and on minimizing the complications related to comorbidities (like epilepsy). New pharmacological treatment options, that target the underlying defect related to each genetic mutation, are currently being explored and may offer opportunities to directly improve cognition [3]. In spite of the emerging interest in cognitive improvement in ID, little is known about patients’ ability to improve core ID symptoms as a response to placebo treatment. We decided to examine to what extent placebo treatment in randomised controlled trials (RCTs) led to clinical improvement in patients with ID. As current theories suggest that placebo effects depend on cognitive inferences based on prior experience (learning) or treatment expectations (reasoning) [4], the severe cognitive deficits in ID may challenge existing placebo models.

In clinical trials, placebo effects can be substantial and are often attributed to the expectations formed at the beginning of the trial, as studies show that the information content may shape placebo outcomes [5]. Yet, little is known about the placebo response in individuals with limited ability to understand the significance of participating in a clinical trial. This is noteworthy, as the investigation of placebo responses in patients with ID may (i) elucidate the impact of the “provision of care” independent of active medication [6], (ii) revise accepted theories of placebo mechanisms that focus on learning and higher order cognition [4, 7, 8], and (iii) improve the way clinical trials are performed and interpreted [9].

The brain mechanisms associated with placebo responses have been described in studies of Parkinson disease [10, 11], pain [12–15], depression [16] and anxiety [17, 18] and suggest a model of prefrontal cognitive control that modulates activity and neurotransmitter function further down the neural axis. Even if subcortical structures [11, 17, 19], and even spinal structures [20, 21], have been associated with placebo responses, the role of executive brain function is considered critical for processing the treatment expectations that create placebo responses. However, recent evidence from our group suggest that human placebo mechanisms can operate outside of conscious awareness [22, 23], challenging the role of higher-order cognitions and calling for closer investigation and refinement of the role of intellectual capacity in theories of placebos.

Here, we used data from RCTs focusing on core ID symptoms (behavioral or cognitive developmental functions) to investigate if patients with genetically determined ID display placebo responses. To address the critical question of report bias, we compared the placebo response to subjective (a third person’s evaluation of the patient, e.g., clinicians’ or parents’ ratings) with more objective outcome measures (direct evaluation of the patient’s abilities, e.g., motor or memory task). By limiting our investigation to genetically determined syndromes we controlled for the possibility that acquired ID (e.g. from childhood disease) could be an advantage due normal brain development before ID onset. There is always a possibility that the placebo response is partially represented by spontaneous remission, or the natural history of the condition being treated. Yet, genetically determined ID is a refractory condition [2], with little likelihood of improvement due to spontaneous remission, rather than an effect of the treatment intervention. Thanks to progress in molecular genetics, and a better knowledge about the physiopathology of each disease, new avenues are emerging for treatment of genetically determined ID, adding to the importance of understanding the placebo response in ID clinical trials.

## Methods

### Data source

We adhered to the PRISMA guidelines for reporting meta-analyses (www.prisma-statement.org). We searched Medline/PubMed, EMBASE, CENTRAL and PsycINFO and restricted our search to randomized placebo-controlled trials in patients with genetically determined ID from one of the following diagnoses: Fragile X (FX), Down’s (DS), Prader-Willi’s (PW), and Williams’ syndromes. The search terms ‘Randomized controlled trial’, ‘Clinical Trial’, ‘Therapeutics’ AND ‘Fragile X syndrome’, ‘Down’s syndrome’, ‘Prader-Willi’s syndrome’, ‘Williams syndrome’ were used. All studies published before April 2013 were considered for inclusion.

### Study selection

The criteria for inclusion were randomized double-blind placebo-controlled trials in patients with one of the *a priori* defined genetically determined syndromes of ID, of any age. Studies were excluded if (i) *outcomes* were not evaluation of behavioral or cognitive-developmental functions (e.g. outcomes such as weight, height), (ii) if the *treatment* targeted a peripheral comorbidity to intellectual disability (e.g. bone mass, acute lymphoblastic leukemia, supra-gingival plaque accumulation). The selection of studies followed the Consolidated Standards Of Reporting Trials (CONSORT) guidelines to ensure adequate quality of included studies (www.consort-statement.org).

### Data extraction and quality assessment

Eligibility judgment of studies was performed in consensus meeting before extraction of data. Studies were excluded if (i) the studies did not report any inference test or enough descriptive information to compute an effect size (for instance, percentages or means and a variability measure), (ii) less than five patients were included, (iii) no separate reports for drug/placebo were available, (iv) not a randomized trial (placebo group added from previous study), (v) only healthy controls were treated and no patients. All data was extracted independently by two separate reviewers (KY, KJ). Once all data was extracted, the values were compared and inconsistencies were resolved in consensus meetings and confirmed with a third reviewer (AC).

For each study, we extracted outcome data for the placebo and drug treatment arms separately, both at baseline and at study end. Each outcome was categorized as either an objective (direct measurement of the patient’s abilities, e.g. a motor or memory task) or subjective measure (a third person’s evaluation of the patient, e.g. a clinician’s rating of symptom severity). Moreover, each outcome was classified according to one of seven mental processes, or domains: attention/executive function, language, memory, cognitive-developmental level, autistic traits, abnormal behavior, and Global Improvement. Measured characteristics of participants included diagnosis, physical age, IQ-level, proportion of men and women and presence of comorbid dementia. Design characteristics included design type, intervention drug(s), country of study origin, treatment duration, number of treatment intakes daily, and year of publication.

### Data synthesis and analysis

Data management, and calculation of bias-corrected standardized mean difference (Hedges’s *g*) were performed using the Comprehensive Meta-Analysis software version 3.0 (www.meta-analysis.com).

Since considerable heterogeneity was expected, all analyses were performed with random-effects rather than a fixed-effects model [24]. The reported Q statistic reflects the distance of each study from the mean effect (weighted, squared, and summed overall studies). Additional sensitivity analyses explored the effect of bias on the results. First, we assessed the presence of publication bias visually by Funnel plot and formally by its statistical analogues, the Begg and Mazumdar-rank correlation test. Sensitivity to the estimate of publication bias was assessed by the Trim and Fill method.

Two types of analyses for placebo and drug effects were performed: one assessing the effect size of pre-post treatment within each treatment arm (drug and placebo separately), the other assessing the effect size between drug and placebo within each study. When a study provided several outcome measures, all of them were combined to provide one value per study.

A priori defined subgroups of data were analyzed by mixed-effects analyses, using the weighted mean effect size for each group (based on random-effect weights), to test the difference between these values (Q statistic). The subgroups were: patients with co-morbid dementia/patients with no dementia, subjective/objective outcomes, type of diagnosis (FX, DS, PW) and drug category (Ampakine, anorexic drug, GABA agonist, Growth Hormone, Inhibitor of acetylcholine esterase, NMDA antagonist, tetracycline, thyroxine, aminoacid, vitamins).

The relationship between pre-defined continuous variable moderators (age, IQ, year of publication and trial duration) and effect size was analyzed using meta-regression, as implemented in the Comprehensive Meta-Analysis software.

## Results

### Description of studies

**Fig 1:**
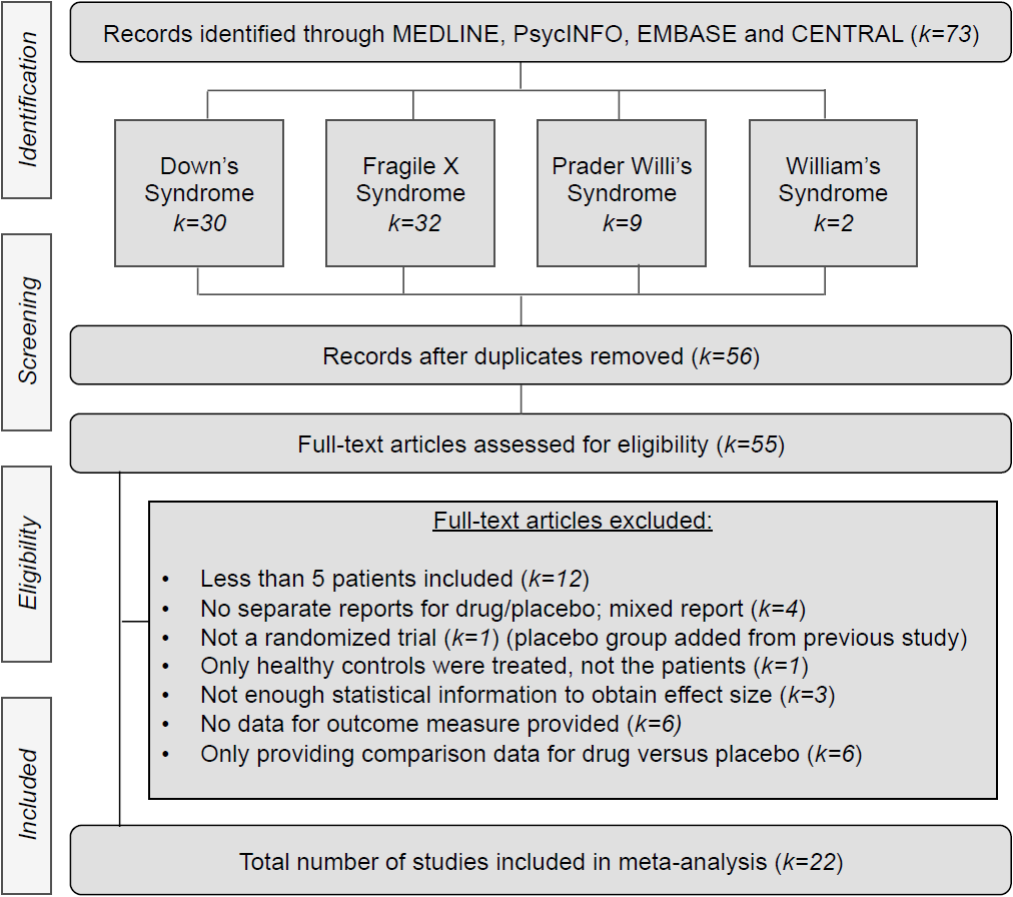
Trial flow chart.

The total number of studies that met the inclusion criteria and were included in the final analyses were *k* = 22 (DS *k* = 14; FX *k* = 6; PW *k* = 2; Williams syndrome *k* = 0), comprising 721 patients in the placebo arm. Fig 1 shows the trial flow and Table 1 provides descriptive features of the studies. The mean duration of studies was 35 weeks (SD 37, range 4–156 weeks). 62% of the participants were male, and the mean age in the placebo arm was 17.1 (SD 15.6, range 0–55) and 17.8 (SD 16, range 0–53) in the drug arm. Studies were conducted in North America (k = 13), Europe (k = 6), Asia (k = 2), and Australia (k = 1).

**Table 1:** Study characteristics. Descriptive features of the studies included in the placebo controlled trials of the meta-analysis.

Study name	Diagn.	Placebo arm			Drug arm			Male (%)	Study design	Type of drug	Trial duration (weeks)
		No.of patients N	Age Mean	Age SD	No. of patients N	Age Mean	Age SD				
**Bennett, 1983 [25]**	DS	10	10	3	10	11	2	50	Parallel	Vitamins**	35
**Berry-Kravis, 2006 [26]**	FX	25	25	8	24	31	10	78	Parallel	Ampakine	4
**Berry-Kravis, 2012 [27]**	FX	56	16	*	56	16	*	87	Crossover	GABA_B_-agonist	4
**Blehaut, 2010 [28]**	DS	44	1	1	43	1	1	53	Parallel	Vitamins**	52
**Boada, 2012 [29]**	DS	19	23	4	18	23	4	37	Parallel	NMDA-antag.	16
**Ellis, 2008 [30]**	DS	33	0	0	106	0	0	57	Parallel	Vitamins**	78
**Hagerman, 1986 [31]**	FX	25	16	10	25	16	10	100	Crossover	Vitamins**	26
**Hanney, 2012 [32]**	DS	74	51	7	72	52	7	57	Parallel	NMDA-antag.	52
**Haqq, 2003 [33]**	PW	12	9	3	12	9	3	50	Crossover	Growth hormone	26
**Johnson, 2003 [34]**	DS	9	25	8	9	30	10	58	Parallel	AChEI	12
**Kishnani, 2009 [35]**	DS	59	26	6	56	24	5	63	Parallel	AChEI	12
**Kishnani, 2010 [36]**	DS	65	13	2	62	13	2	52	Parallel	AChEI	10
**Kondoh, 2011 [37]**	DS	10	44	*	11	47	*	0	Parallel	AChEI	24
**Leigh, 2013 [38]**	FX	53	9	4	50	9	4	85	Crossover	Tetracycline	13
**Prasher, 2002 [39]**	DS	13	55	5	14	53	8	50	Parallel	AChEI	24
**Pueschel, 1980 [40]**	DS	20	0	0	69	0	0	56	Parallel	Vitamins**	156
**Sahu, 2013 [41]**	FX	10	8	3	10	10	3	100	Parallel	AChEI	12
**Selikowitz, 1990 [42]**	PW	15	14	*	15	14	*	[30]	Crossover	Anorexic	6
**Smith, 1984 [43]**	DS	28	11	3	28	11	3	71	Parallel	Vitamins**	35
**Torrioli, 2008 [44]**	FX	27	9.18	*	24	9.18	*	100	Parallel	L-acetyl carnitine	52
**Van Trotsenburg, 2005 [45]**	DS	91	0	0	90	0	0	55	Parallel	Thyroxine	104
**Weathers, 1983 [46]**	DS	23	12	*	24	12	*	66	Parallel	Vitamins**	17
**Placebo controlled studies**		**721**	**17.1**	**15.6**	**828**	**17.8**	**16**	**62**			**35**

* not provided

** We used “vitamins” as a category for studies using either folic acid alone or a combination of vitamins, minerals and/or antioxidants.

Abbreviations: Down Syndrome (DS), Fragile X Syndrome (FX) and Prader-Willi Syndrome (PW). Gamma-Aminobutyric acid (GABA) agonist, N-metyl-D-aspartat (NMDA) antagonist, Acetylcholinesterase Inhibitor (AchEI).

### Placebo and drug response

**Fig 2:**
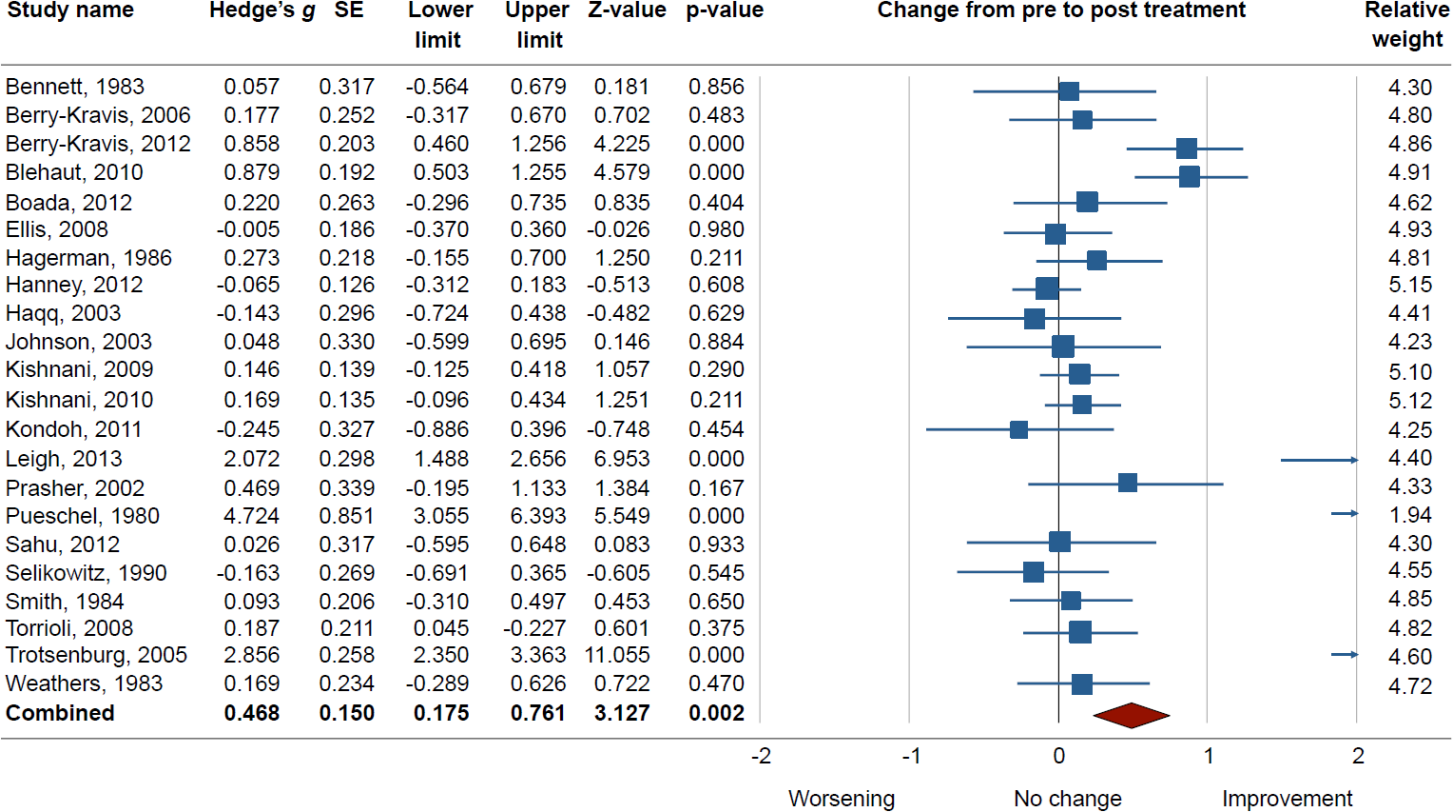
Forest plot of placebo responses in patients with Intellectual Disability. A significant placebo response was found from pre to post treatment (p = 0.002) across all studies. If studies included more than one outcome measure they were combined into one value. A random-effects model was used to calculate significance. There was a medium overall effect size, which is comparable to the placebo effect size in studies with patients who are not intellectually disabled [47, 48].

The pre-post comparison of treatment outcomes in the placebo arm of the included trials showed a significant overall placebo response, based on study effect size (*g* = 0.468, SE = 0.150, p = 0.002, see Fig 2). We identified significant heterogeneity across studies (Q = 201.9, df = 21, p<0.001, I^2^ = 89.59, tau^2^ = 0.41), and attempted to explain this variability by means of subgroup analyses. The same analysis was performed for the drug arm, where a pre-post comparison showed a significant drug response (*g* = 0.678, SE = 0.171, p = 0.0001). A within-study comparison of drug and placebo (traditional drug-placebo meta-analysis) showed a non-significant difference (*g* = 0.061, SE = 0.052, p = 0.24). For both treatment arms, the Funnel plots revealed no evident publication bias. The Begg’s test result was not statistically significant and the Trim and Fill method did not lead to any adjustment of the results for the placebo arm. As there were three studies with clearly higher effect size than the rest ([38, 40, 45]), we tested if the placebo meta-analysis would still be significant with them removed. A random effects analysis (*k* = 19) revealed a significant yet smaller effect size (*g* = 0.2, SE = 0.071, p = 0.01) compared to overall effect size when including all studies (g = 0.468).

### Comparison of subjective and objective outcomes

We compared the placebo response to subjective measures (a third person’s evaluation of the patient, e.g., clinicans or parents’ ratings of severity) with more objective outcome measures (direct evaluation of the patient’s abilities such as motor or memory task) in order to address the question of report bias when responding to placebo treatment. We found significant placebo responses both for subjective (*g* = 0.563, SE = 0.246, p = 0.022) and objective (*g* = 0.434, SE = 0.207, p = 0.036) outcomes. A comparison between objective and subjective outcomes was non-significant (*Q* = 0.161, df = 1, p = 0.688), indicating that placebo responses are not significantly greater for subjective outcomes than for objective measures.

### Effect of mental processes categories

The effect size of the placebo response differed significantly by different mental process categories (*Q* = 13.288, df = 6, p = 0.039). Table 2 shows the results observed for each category. The pre-post comparison for autistic traits, abnormal behavior, cognitive and developmental skills and Clinical Global Improvement showed a significant placebo response. However, no significant placebo response was observed on attention, language and memory. The drug response also differed significantly by mental process categories (*Q* = 17.348, df = 6, p = 0.008). No significant category difference was seen between drug and placebo.

**Table 2:** Placebo and drug responses by mental process categories. The total number of scales used for assessment of treatment outcomes were grouped into 7 different mental process categories, or domains. Four out of seven domains had a significant placebo response and five out of seven domains had a significant drug response. Clinical Global Impression (CGI) assessments are commonly used as study endpoints in clinical trials. Here, CGI is represented by one category, even if it is not a mental process.

	Placebo response			Drug response			Drug / Placebo comparison		
Mental process	*g*	SE	p-value	*g*	SE	p-value	Q	df(Q)	p-value
Attention (*k = 2*)	0.056	0.229	0.81	0.180	0.234	0.441	0.143	1	0.71
Language (*k = 8*)	0.017	0.064	0.79	0.121	0.063	0.05*	1.327	1	0.25
Memory (*k = 4*)	0.174	0.132	0.19	0.282	0.159	0.08	0.272	1	0.60
Cognitive and developmental (*k = 17*)	0.305	0.121	0.01*	0.521	0.137	0.0001****	1.394	1	0.24
Abnormal Behavior (*k = 7*)	0.278	0.09	0.002***	0.480	0.127	0.0001****	1.686	1	0.19
Autistic traits (*k = 3*)	0.336	0.161	0.037*	0.394	0.142	0.006**	0.073	1	0.79
CGI (*k = 3*)	2.215	1.049	0.035*	2.004	0.800	0.01*	0.026	1	0.87

* < .05

** < .01

*** < .005

**** < .001

### Effect of Intelligence level (IQ) on treatment outcomes

Using a regression model, we assessed the effect of IQ (which varied from 20 up to 65) on treatment response and found a significant effect of IQ corrected for age on placebo responses (*Q* = 7.48, df = 2, p = 0.02), represented by larger placebo responses in patients with higher IQ. There was no similar effect for drug response (*Q* = 0.46, df = 2, p = 0.80).

### Effect of age on treatment outcomes

**Fig 3:**
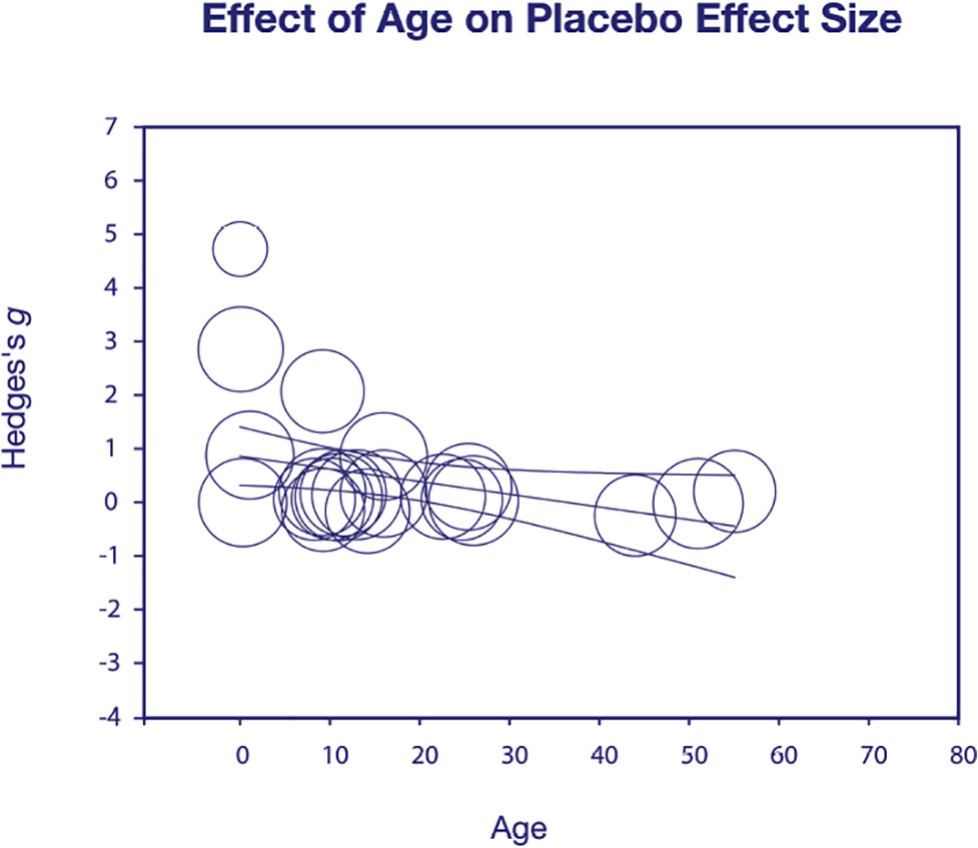
Effect of age on placebo effect size. There was a significant effect of age on placebo effect size (*g*), represented by higher placebo responses in the youngest patients included in this meta-analysis (p < .05). It is possible that the results are explained by stronger placebo-by-proxy effects in young children, as parents and caregivers express high encouragement, attention and support to babies. The intensive care and attention given to young children may wane over time and thus lessen the placebo effects in older ID patients.

There was a significant effect of age on placebo effect size (*Q* = 5.25, df = 1, p = 0.02), indicating that younger patients had larger placebo responses (Fig 3). We found a comparable effect of age on drug effect size (*Q* = 8.56, df = 1, p = 0.0034).

### Effect of dementia on treatment outcomes

Comorbid dementia in patients with ID had a significant effect on placebo responses (*Q* = 4.249, df = 1, p = 0.039), but not on drug responses (*Q* = 1.961, df = 1, p = 0.161). Specifically, patients with no dementia showed robust placebo responses (*g* = 0.508, SE = 0.160, p = 0.002) whereas patients with comorbid dementia had virtually no response to placebo (*g* = -0.050, SE = 0.218, p = 0.82).

### Regression with trial duration

We found a positive effect of trial duration on placebo effect size (*Q* = 16.38, df = 1, p = 0.0001), with results pointing to larger effect sizes with longer trial durations. Two outliers appeared to drive the effect [40, 45] and with them removed the effect of trial duration was non-significant. Nonetheless, removal of these outliers did not affect the observation that the placebo response did not diminish over time. This finding is contrary to the common belief that placebo responses would be short lasting and wane with time [49].

### Effect of diagnosis category

The effect size of the placebo response differed significantly between diagnostic categories (*Q* = 7.367, df = 2, p = 0.025). Placebo responses were observed both in DS (*g* = 0.510, SE = 0.198, p = 0.01) and FX (*g* = 0.590, SE = 0.273, p = 0.03), but not in PW patients (*g* = -0.154, SE = 0.199, p = 0.44). The number of studies included in the PW group was likely too small (k = 2) to allow enough statistical power to this analysis. Conversely, the effect size of the drug response did not significantly differ by diagnosis category (*Q* = 3.722, df = 2, p = 0.16).

### Regression with year of publication

The possible effects of time on the development of drugs with high efficacy, and the associated increase in treatment expectations conveyed to patients and their families, was assessed by a regression of treatment effect size and publication year, ranging from 1980 to 2013. We did not find any effect of the year of publication and effect size, neither in placebo (*Q* = 0.06, df = 1, p = 0.81), nor drug (*Q* = 1.34, df = 1, p = 0.25).

### Effect of drug category

A regression model revealed that the effect size of both placebo and drug responses differed significantly by drug category (placebo *Q* = 158.243, df = 9, p = 0.0001 and drug *Q* = 144.433, df = 9, p = 0.0001 respectively). Unpacking the overall regression model shows that trials involving either a GABA agonist, Inhibitor of acetylcholine esterase, tetracycline, thyroxine, L-acetyl carnitine or vitamins had a significant drug response. Among them, the trials involving an inhibitor of acetylcholine esterase did not show a comparable placebo response, possibly related to the presence of patients with comorbid dementia in these studies.

## Discussion

To our knowledge, this meta-analysis presents the first account of placebo responses in ID patients and the first evaluation of the role of IQ in placebo mechanisms. Here, we demonstrate that patients with ID display significant placebo responses in spite of severe impairment of mental abilities such as learning, reasoning, abstract thinking and predicting the future. The overall effect size of placebo responses in this study (*g* 0.5) is considered a “medium” effect and is comparable to the placebo response in adult patients without ID [47, 48] and in children [50] across a range of domains such as migraine, depression, Attention Deficit Hyperactivity Disorder and osteaoarthritis. Previous meta-analyses have used the same approach to studying placebo responses in different clinical populations, using data from the placebo arm of RCTs in e.g. depression [51], irritable bowel syndrome [52], restless legs [53], and schizophrenia [54]. The strong evidence for placebo responses in ID, illustrated both in subjective and more objective outcomes, and in a wide range of behavioral and cognitive domains, challenges current theories of placebo mechanisms and suggests possibilities for change in the delivery of care. The fact that there was no significant difference in placebo effect size between subjective and objective outcome measures suggests that placebo responses in ID is not an effect of report bias, but likely represents a true improvement of patients’ symptoms. Conversely, if placebo effects had mainly been present in response to subjective reports (given by caretakers with no intellectual deficiency) one could not have concluded that individuals with ID display placebo responses. Given the refractory nature of genetically determined ID, it seems reasonable to assume that the improvements in response to placebos are primarily related to the provision and context of care, and not to natural remission of ID symptoms. This suggests that a supportive and attentive environment may lead to improvements in patients with ID.

The traditional drug versus placebo meta-analysis did not reveal any significant difference between drug and placebo. It is interesting to note that, we found a significant drug response from baseline in the drug arm. It is likely that this drug effect, which reflects both drug effect per se and placebo responses, is primarily related to placebo responses we observed in these studies.

### Possible mechanisms of placebo responses in ID

This meta-analysis only included genetically determined ID, i.e. a condition that existed from conception, thus ensuring that the observed placebo response was not confounded by patients with a normal brain development before onset of ID. One key mechanism of placebo responses, described in individuals without ID, involves *conscious expectancy* [55]. The content of information related to treatment, such as referring to a topical placebo cream as “inert and with no effect” or as a “powerful painkiller” [56], can manipulate expectation and mediate placebo effects. Yet, it seems unlikely that the explicit information content play a major role in generating placebo responses in patients with ID, given their specific reasoning and language difficulties (both in comprehension and production).

Placebo effects can also be induced by mechanisms that are automatic and operate on a non-conscious level, such as *associative learning* (conditioning) [57]. Placebo responses have also been demonstrated in treatment of animals [58, 59]. In a previous study we challenged the exclusive role of conscious cognitions in human placebo responses and suggest that treatment cues may be implicitly embedded in the patient–clinician interaction and clinical environment [22, 23]. It is likely that non-conscious placebo mechanisms may play a role in patients with ID, as they involve learning that does not rely on abstraction.

Closely related to non-conscious placebo effects, and a third possible mechanism, is the implicit social influence of “*placebo by proxy*” [60], which has been mentioned apropos of placebo effects in young children. Placebo by proxy can operate in two ways. It might produce a genuine change in the patient’s condition, and/or it might alter the parent or caregiver’s perception of the patient’s behavior [61]. The significant placebo response we observed on subjective outcomes (evaluation of the patient by a third person) are likely related to the latter. Grelotti and Kaptchuk argue that “*if clinicians and family members feel empowered and optimistic about a treatment, they may smile more, pay more attention to the patient, promote treatment adherence, encourage the patient to engage in new activities etc” [60].* In this way, Grelotti and Kaptchuk suggest that placebo by proxy changes the patient’s psychosocial context, and mediates the placebo effect in a way that is not strictly a learning phenomenon or conscious expectancy; yet it may have the same profound influence on patient’s physiology. Placebo by proxy has also been observed in children with tantrums [61], autism [62], cerebral palsy and Attention-Deficit/Hyperactivity Disorder (ADHD) [63] and it is likely that this mechanism play a major role in ID trials, as ID patients are very sensitive to changes in their environment [64–66]. In the case of placebo by proxy, it is the treatment expectations of the clinicians and family members that elicit a placebo effect on the ID patient mediated by altered behavior toward him.

Contrary to our findings, a previous study speculated that ID patients would not display placebo responses, as there is evidence for loss of placebo responses in patients with dementia (due to impaired cognitive functioning) [67]. This furthers the notion that patients with genetically determined ID may have developed an *alternate mechanism* for placebo responses that relies on an early compensatory adaption. As ID patients are born with cognitive impairments, it is likely that adaptive responses to the challenges in the environment occur with time. In dementia however, already established response patterns are disrupted, leaving the patient with severe cognitive disabilities. Along these lines, the placebo responses in ID patients could be described as a “low road” adaptation, which is not related to higher-order cognitive function (possibly through patients’ enhanced sensitivity to contextual and social cues), compared to the “high road” predictions and expectations described in healthy individuals.

### IQ

Even if patients with ID display robust overall placebo responses, there was a significant effect of IQ on placebo effect size (but not on drug effect size). It is possible that high-functioning ID patients have a normal placebo response, but at a certain degree of intellectual impairment there is no longer a response. Since a limited number of clinical trials reported IQ levels for their patients, our findings should be confirmed in future analysis where the relationship between IQ and placebo responses can be further explored. We found that ID patients displayed significant placebo responses before, but not after, onset of dementia, confirming the previous mentioned study suggesting decreased placebo responses related to severe loss of cognitive skills in patients with dementia [67].

### Trial duration

Placebo effects are anecdotally thought to have a short duration, as they rely on learning mechanisms that may wane with time [49]. Here, we found that the placebo responses were stable over time, in spite of trials lasting up to three years. This is in line with the stable placebo effects seen in various long lasting clinical trials [68, 69]. We hope that future studies will investigate the long-term effects of placebo treatment in ID patients.

### Syndrome-related specificities

We found placebo responses both in DS and FX patients, but cannot conclude anything about PW given the small number of studies. DS and FX patients both exhibit a moderate to severe range of ID but have different social and affective profiles. DS patients are frequently reported to demonstrate empathy and care for others, showing concern and offering comfort to the person in distress [70], while FX patients display social hyper-arousal, which leads to gaze aversion, social difficulties and anxiety [71, 72]. In both syndromes, ID patients usually would like to please their doctor and caretaker [73]. Both DS and FX patients are sensitive to surrounding cues of expectancy, even if it may be from different disorder-specific abilities (e.g. good social and communicative skills in DS; and lack of inhibition or filtering of the environment in FX) [74–77].

### Age

We found a significant relationship between age and placebo effect size, with higher placebo responses in very young children. Four trials included children at birth and it is likely that the high placebo response is an effect of a particularly intensive placebo by proxy in newborns. A recent review suggests an inverse relationship between age and placebo effect size in healthy children [50], validating our findings in patients with ID. Children tend to assume that they are receiving real medication more often than adults [78] and meta-analyses across several medical disciplines report robust placebo responses in children [50, 79–81].

### Placebo in different cognitive and behavioral domains

The placebo effect differed significantly by mental process categories. Even if this could partly be an effect of the different scale sensitivities, it is possible that placebo responses are not equally expressed across domains. Many different scales were used in the different clinical trials included here. Future work is necessary (as suggested by the recent meeting convened by the National Institutes of Health, Washington DC, 2012) in order to identify reliable and sensitive outcome measures for common use in clinical trials in FX [82] and more generally in ID patients.

### Limitations and future perspectives

As previously mentioned, there is always a possibility that the placebo response is partially represented by spontaneous remission, or the natural history of the condition being treated. However, this study only includes patients with genetically determined ID, which is a condition without spontaneous remission [2]. Nevertheless, even if the intellectual disability will not improve over time, some comorbid symptoms may fluctuate over time, such as tantrums, hyperactivity or agitation [73]. As some of them may be included as outcome measures to evaluate the behavior of the patients, it is likely that these outcomes may be more sensitive to placebo too. If future pharmacological trials include a natural history control arm, in addition to the traditional placebo control arm, there can be a better estimation of the possible contributions of bias to the placebo response.

No significant drug versus placebo effect was observed in our meta-analysis. Yet, novel and hopefully more efficient treatment options for ID are beginning to appear for a number of specific conditions, such as DS and FX [3]. These pathophysiologically targeted treatments are focusing on correcting the underlying defect, via re-equilibration of the biochemical imbalance that results from genetic mutations, instead of suppressing only some of the most pervasive symptoms [83]. Some preliminary results have been promising [84] and raised the expectancy in patient’s families. Given the recent example of secretine trials in children with autism [62, 63], showing robust and consistent reductions in symptoms, but no difference between secretine over placebo, it is necessary to be vigilant on the role of placebos when testing novel treatments in ID.

## Supporting Information

S1 File PRISMA 2009 checklist. (DOCX)
